# Collaboration of a Detrimental HLA-B*35:01 Allele with HLA-A*24:02 in Coevolution of HIV-1 with T Cells Leading to Poorer Clinical Outcomes

**DOI:** 10.1128/JVI.01259-21

**Published:** 2021-11-09

**Authors:** Nozomi Kuse, Hayato Murakoshi, Tomohiro Akahoshi, Takayuki Chikata, Katherine L. James, Hiroyuki Gatanaga, Sarah L. Rowland-Jones, Shinichi Oka, Masafumi Takiguchi

**Affiliations:** a Tokyo Laboratory and Division of International Collaboration Research, Joint Research Center for Human Retrovirus Infection, Kumamoto University, Kumamoto, Japan; b Center for AIDS Research, Kumamoto University, Kumamoto, Japan; c Nuffield Department of Medicine, University of Oxford, Headington, Oxford, UK; d AIDS Clinical Center, National Center for Global Health and Medicine, Tokyo, Japan; Emory University

**Keywords:** CD8, CTL, HIV-1, HLA, TCR, escape mutation

## Abstract

Although mutant-specific T cells are elicited in some individuals infected with HIV-1 mutant viruses, the detailed characteristics of these T cells remain unknown. A recent study showed that the accumulation of strains expressing Nef135F, which were selected by HLA-A*24:02-restricted T cells, was associated with poor outcomes in individuals with the detrimental HLA-B*35:01 allele and that HLA-B*35:01-restricted NefYF9 (Nef135-143)-specific T cells failed to recognize target cells infected with Nef135F mutant viruses. Here, we investigated HLA-B*35:01-restricted T cells specific for the NefFF9 epitope incorporating the Nef135F mutation. Longitudinal T-cell receptor (TCR) clonotype analysis demonstrated that 3 types of HLA-B*35:01-restricted T cells (wild-type [WT] specific, mutant specific, and cross-reactive) with different T cell repertoires were elicited during the clinical course. HLA-B*35:01^+^ individuals possessing wild-type-specific T cells had a significantly lower plasma viral load (pVL) than those with mutant-specific and/or cross-reactive T cells, even though the latter T cells effectively recognized the mutant virus-infected cells. These results suggest that mutant-specific and cross-reactive T cells could only partially suppress HIV-1 replication *in vivo*. An e*x vivo* analysis of the T cells showed higher expression of PD-1 on cross-reactive T cells and lower expression of CD160/2B4 on the mutant-specific T cells than other T cells, implying that these inhibitory and stimulatory molecules are key to the reduced function of these T cells. In the present study, we demonstrate that mutant-specific and cross-reactive T cells do not contribute to the suppression of HIV-1 replication in HIV-1-infected individuals, even though they have the capacity to recognize mutant virus-infected cells. Thus, the collaboration of HLA-A*24:02 with the detrimental allele HLA-B*35:01 resulted in the coevolution of HIV-1 alongside virus-specific T cells, leading to poorer clinical outcomes.

**IMPORTANCE** HIV-1 escape mutations are selected under pressure from HIV-1-specific CD8^+^ T cells. Accumulation of these mutations in circulating viruses impairs the control of HIV-1 by HIV-1-specific T cells. Although it is known that HIV-1-specific T cells recognizing mutant virus were elicited in some individuals infected with a mutant virus, the role of these T cells remains unclear. Accumulation of phenylalanine at HIV-1 Nef135 (Nef135F), which is selected by HLA-A*24:02-restricted T cells, led to poor clinical outcome in individuals carrying the detrimental HLA-B*35:01 allele. In the present study, we found that HLA-B*35:01-restricted mutant-specific and cross-reactive T cells were elicited in HLA-B*35:01^+^ individuals infected with the Nef135F mutant virus. These T cells could not effectively suppress HIV-1 replication *in vivo* even though they could recognize mutant virus-infected cells *in vitro*. Mutant-specific and cross-reactive T cells expressed lower levels of stimulatory molecules and higher levels of inhibitory molecules, respectively, suggesting a potential mechanism whereby these T cells fail to suppress HIV-1 replication in HIV-1-infected individuals.

## INTRODUCTION

HIV-1-specific CD8^+^ T cells play an important role in the control of HIV-1 (1–6). Some of these T cells select HIV-1 escape mutants, leading to the accumulation of escape mutant viruses in populations and the adaptation of HIV-1 to the host immune system (7–11). It is known that most of these mutations are associated with specific HLA class I alleles (12–17). Several hundred HLA-associated mutations have been reported in HIV-1 subtype B, C, and A/E infections, although only some of these HLA-associated mutations have been confirmed functionally as being T-cell escape mutations. Since escape mutations usually impair the recognition of target cells infected with mutant viruses by HIV-1-specific T cells, the accumulation of escape mutant viruses in the population results in poorer clinical outcomes in patients with HIV-1 infection (7, 18, 19). The existence of escape mutations among reservoir and circulating viruses is a critical barrier for the eradication of latent HIV-1 reservoirs and the prevention of HIV-1 infection.

Escape mutations critically affect T-cell receptor (TCR) recognition, epitope peptide binding to HLA molecules, and/or the generation of the epitope peptide in HIV-1-infected cells (17). Therefore, escape mutant-specific T cells are elicited rarely in individuals infected with escape mutant viruses. Recent studies reported some cases in which T cells specific for HIV-1 escape mutations or HLA-associated mutations were elicited in individuals infected with viruses expressing an escape- or HLA-associated mutation (11, 18, 20–25). However, these T cells show no or a very limited ability to suppress the replication of mutant viruses *in vitro* (7, 11, 19), suggesting that these T cells would also not be able to suppress HIV-1 replication *in vivo*. Since the generation of mutant-specific T cells and an analysis of their function have been investigated only partially to date, the role of these T cells in HIV-1 infection remains unclear.

HLA-B*35 is associated with a rapid progression to AIDS and poor clinical outcomes (26–31). HLA-B35 subtypes, such as HLA-B*35:02 and B*35:03, which have the peptide binding motif of proline at P2 and a nontyrosine residue at the C terminus, referred to as HLA-B*35-PX, have a significant association with the rapid AIDS progression in Caucasians, whereas other subtypes with the motif proline at P2 and tyrosine at the C terminus (HLA-B*35-PY) do not (27, 28). On the other hand, a large-scale cross-sectional study in a European cohort infected with HIV-1 clade B demonstrated that among HLA-B*35:01^+^ individuals, the frequency of HIV-1 controllers was significantly lower than that of progressors (29). Several mechanisms to explain the detrimental effect of HLA-B*35 have been proposed (31–33). They include the loss of HLA-B*35-restricted T cells specific for a Gag epitope (31); impaired dendritic cell function via an inhibitory major histocompatibility complex (MHC) class I receptor, ILT4 (32); and dysfunction of CD8^+^ T cell via selective upregulation of CTLA-4 (33).

Our recent study suggested that the Nef135F mutation, which is selected by HLA-A*24:02-restricted T cells specific for RF10 (Nef134-143: RYPLTFGWCF) and/or RW8 (Nef134-141: RYPLTFGW), is a key factor underlying the detrimental effect of HLA-B*35:01 on disease outcomes in HIV-1 clade B-infected individuals (34). This study showed that HLA-B*35:01-restricted T cells specific for NefYF9 (Nef135-143; YPLTFGWCF) failed to recognize target T cells infected with Nef135F mutant viruses, implying that these T cells could not suppress the replication of the Nef135F mutant virus *in vivo*. In addition, the accumulation of Nef135F was associated with poor clinical outcomes among the HLA-B*35:01^+^ individuals (34). On the other hand, the binding affinity of the YF9 peptide for HLA-B*35:01 is almost identical to that of the FF9 mutant peptide (34), suggesting the possibility that FF9-specific T cells could be elicited in HLA-B*35:01^+^ individuals infected with the Nef135F mutant virus. However, it remains unknown whether HLA-B*35:01-restricted T cells specific for the NefFF9 mutant epitope are elicited in individuals infected with the mutant virus; similarly, the role of mutant-specific T cells *in vivo* in HIV-1 infection is not clear.

In the present study, we analyzed how FF9 mutant-specific and cross-reactive CD8^+^ T cells were elicited in HLA-B*35:01^+^ individuals infected with the Nef135F mutant virus. We further investigated the coevolution of HIV-1 with those T cells recognizing the mutant FF9 epitope and looked at any potential association of the presence of mutant-specific T cells with clinical outcome among HIV-1-infected HLA-B*35:01^+^ individuals. These analyses should clarify the role of escape mutant-specific T cells and cross-reactive T cells during HIV-1 infection.

## RESULTS

### HLA-B*35:01-restricted CD8^+^ T cells specific for the FF9 mutant epitope are elicited in an HLA-B*35:01^+^ individual infected chronically with the HIV-1 Nef135F mutant virus.

**FIG 1:**
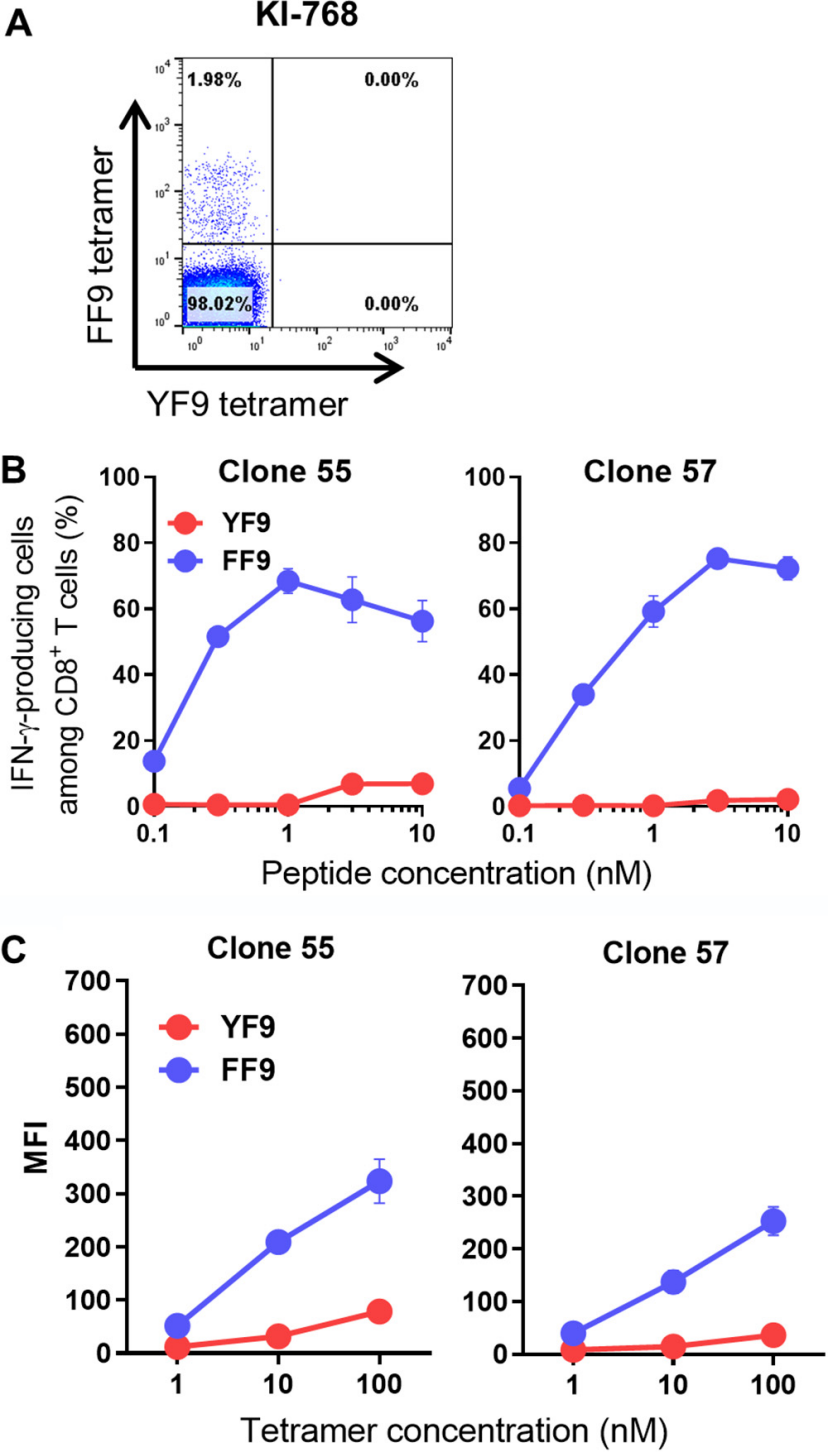
Elicitation of NefFF9 mutant epitope-specific HLA-B*35:01-restricted CD8^+^ T cells in an HIV-1 Nef135F mutant virus-infected HLA-B*35:01^+^ individual. (A) Identification of NefFF9-specific T cells in PBMCs from patient KI-768. PBMCs were stained with anti-CD3 MAb, anti-CD8 MAb, HLA-B*35:01-YF9 tetramer, and HLA-B*35:01-FF9 tetramer. A 100 nM concentration of each tetramer was used to stain PBMCs. The CD8^+^CD3^+^ population was analyzed for tetramer binding. (B) Recognition of YF9 and FF9 peptides by 2 FF9-specific T-cell clones established from patient KI-768. Responses of the T-cell clones to 721.221-B*35:01 cells prepulsed with the YF9 or FF9 peptide were analyzed using the ICS assay. (C) Binding affinity of YF9- or FF9-HLA-B*35:01 tetramers to 2 clones. The binding of these tetramers to the clones was measured at 3 different concentrations (1, 10, and 100 nM).

To clarify whether FF9 mutant-specific HLA-B*35:01-restricted CD8^+^ T cells were elicited in HLA-B*35:01^+^ individuals infected chronically with HIV-1 NefY135F mutant viruses, we analyzed peripheral blood mononuclear cells (PBMCs) from one subject, namely, KI-768, who had been infected with the NefY135F mutant virus, by performing flow cytometry analysis using tetramers specific for both the wild-type and mutant epitopes, i.e., HLA-B35:01-YF9 and HLA-B35:01-FF9 (YF9-tet and FF9-tet, respectively). Our data showed that only FF9-specific CD8^+^ T cells were present in this individual (Fig. 1A). We next established 2 FF9-specific CD8^+^ T-cell clones from this individual. Both T-cell clones effectively recognized 721.221-B3501 cells prepulsed with the FF9 peptide but not those prepulsed with the YF9 peptide (Fig. 1B). These clones bound to FF9-tet but very weakly to YF9-tet only at a high concentration of the tetramer (Fig. 1C). These results together confirmed that HLA-B*35:01-restricted FF9 mutant-specific CD8^+^ T cells were elicited in patient KI-768.

### Longitudinal analysis of HLA-B*35:01-restricted CD8^+^ T cells specific for the YF9/FF9 epitope in an HIV-1-infected HLA-A*24:02/B*35:01^+^ individual.

**FIG 2:**
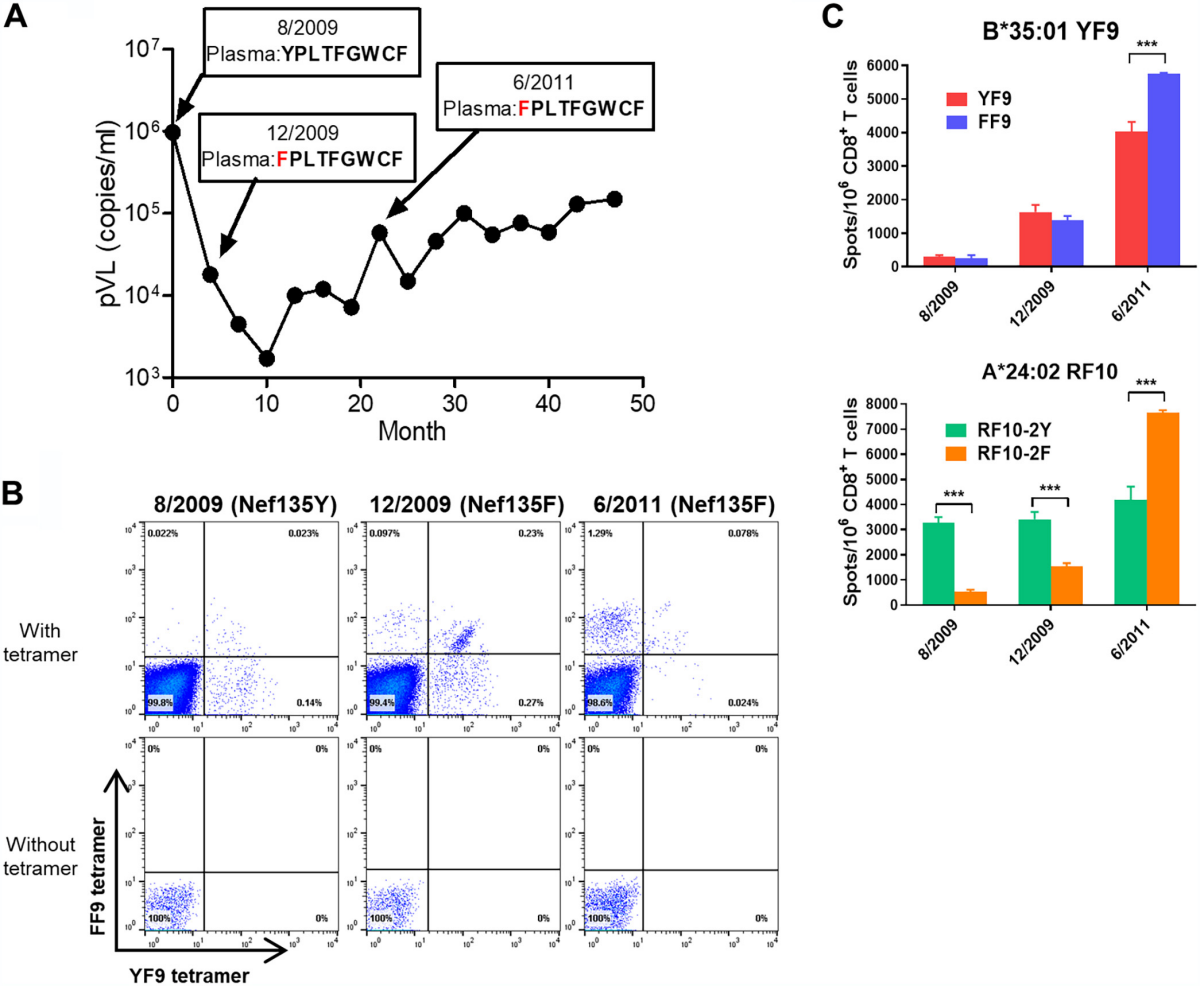
Longitudinal analysis of CD8^+^ T cells specific for the YF9 or FF9 epitope in an HLA-B*35:01^+^ individual who had been infected with the Nef135Y wild-type virus at an early phase and with the Nef135F mutant virus at both early and later phases. (A) Time course of pVL and amino acid sequence of Nef135-143 in patient KI-705. (B) Longitudinal change in the specificity of the HLA-B*35:01-restricted CD8^+^ T cells specific for the YF9/FF9 epitope in KI-705. A population of YF9-specific or FF9-specific CD8^+^ T cells were determined by staining *ex vivo* PBMCs from KI-705 with both HLA-B35:01-YF9 and HLA-B35:01-FF9 tetramers (top) and by staining them without tetramers as a negative control (bottom). (C) Longitudinal changes in the *ex vivo* T-cell responses of KI-705 to the HLA-B*35:01-restricted YF9 or FF9 peptide and to the HLA-A*24:02-restricted RF10-2Y or RF10-2F peptides. The responses to these peptides were assessed using ELISPOT assays. The data are presented as the mean and SD (*n* = 3). Statistical analyses were performed using the unpaired *t* test. ***, *P* < 0.001.

We next performed a longitudinal analysis of HLA-A*24:02-restricted NefRF10-specific and HLA-B*35:01-restricted NefYF9/FF9-specific CD8^+^ T cells in an HLA-A*24:02^+^HLA-B*35:01^+^ individual, namely, KI-705 (Fig. 2A). This individual had been infected with the wild-type Nef135Y virus in August 2009 (during an early phase of the wild-type virus infection), while thereafter the Y135F mutant was the dominant sequence at both subsequent time points, i.e., December 2009 (the early phase of the mutant virus infection) and June 2011 (the chronic phase of the mutant virus infection). We first analyzed the emergence of YF9/FF9-specific CD8^+^ T cells by performing dual staining analysis using both YF9-tet and FF9-tet. At the first time point (August 2009), YF9-specific CD8^+^ T cells were the predominant population, although a few cross-reactive T cells were also detected (Fig. 2B, left). At the second point (December 2009), cross-reactive T cells had become more numerous (approximately 38% of total tetramer-positive CD8^+^ T cells), while YF9-specific T cells were found in approximately 45% of them. A new population of T cells specific for the FF9 mutant epitope (approximately 16% of total tetramer^+^ CD8^+^ T cells) (Fig. 2B, middle) had emerged. At the last time point (June 2011), FF9-specific T cells were the dominant population (Fig. 2B, right). Thus, the specificity of the epitope-specific CD8^+^ T cells changed dramatically after the emergence of the mutant virus.

A small population of cross-reactive CD8^+^ T cells was detected at the first time point. This finding implies the possibility that HIV-1 variants with the escape mutation may have been present as a minor population within the quasispecies at an early time point. To clarify this possibility, we performed RNA deep sequencing to determine whether any of the infecting HIV-1 strains at the first time point harbored the Nef135 mutation. The RNA sequence analysis demonstrated that all of the 89 reads at nucleotide position 9200 contained adenosine, indicating the presence of tyrosine at Nef135 in all sequences analyzed (data not shown). These data argue strongly against the possibility that the Nef135F mutant virus had arisen at the first time point. Taken together, these results support our contention that the few cross-reactive and the wild-type-specific CD8^+^ T cells were both elicited initially by antigen presentation of the wild-type virus.

As we speculated that the Y135F mutation had been selected by HLA-A*24:02-restricted NefRF10-specific CD8^+^ T cells in this individual, we analyzed the CD8^+^ T-cell response to the HLA-A*24:02-restricted epitope peptide RF10 and the relevant peptide variant at 3 time points by performing enzyme-linked immunosorbent spot assay (ELISPOT) assays. At the first time point, T-cell responses to the HLA-B*35:01-restricted YF9 epitope peptide were not detected, whereas T-cell responses to the HLA-A*24:02-restricted wild-type epitope peptide (RF10-2Y) were strongly detected (Fig. 2C). This result indicates that these HLA-A*24:02-restricted RF10-2Y-specific T cells had been elicited earlier than the HLA-B*35:01-restricted YF9-specific T cells. T-cell responses to the HLA-A*24:02-restricted RF10-2F mutant peptide were found at the second time point (only 4 months after the first time point), whereas the response to this peptide was not detected at the first time point (Fig. 2C). These results support the idea that the Y135F mutation had been selected by HLA-A*24:02-restricted RF10-2Y-specific CD8^+^ T cells rather than by HLA-B*35:01-restricted YF9-specific T cells. At the third and last time point, RF10-2F-specific CD8^+^ T cells were the dominant population, as previously reported (34). Thus, this mutation elicited both HLA-B*35:01-restricted T cells and HLA-A*24:02-restricted CD8^+^ T cells specific for the mutant epitopes.

### Longitudinal TCR clonotype analysis of HLA-B*35:01-restricted CD8^+^ T cells specific for the YF9 or FF9 epitope.

**FIG 3:**
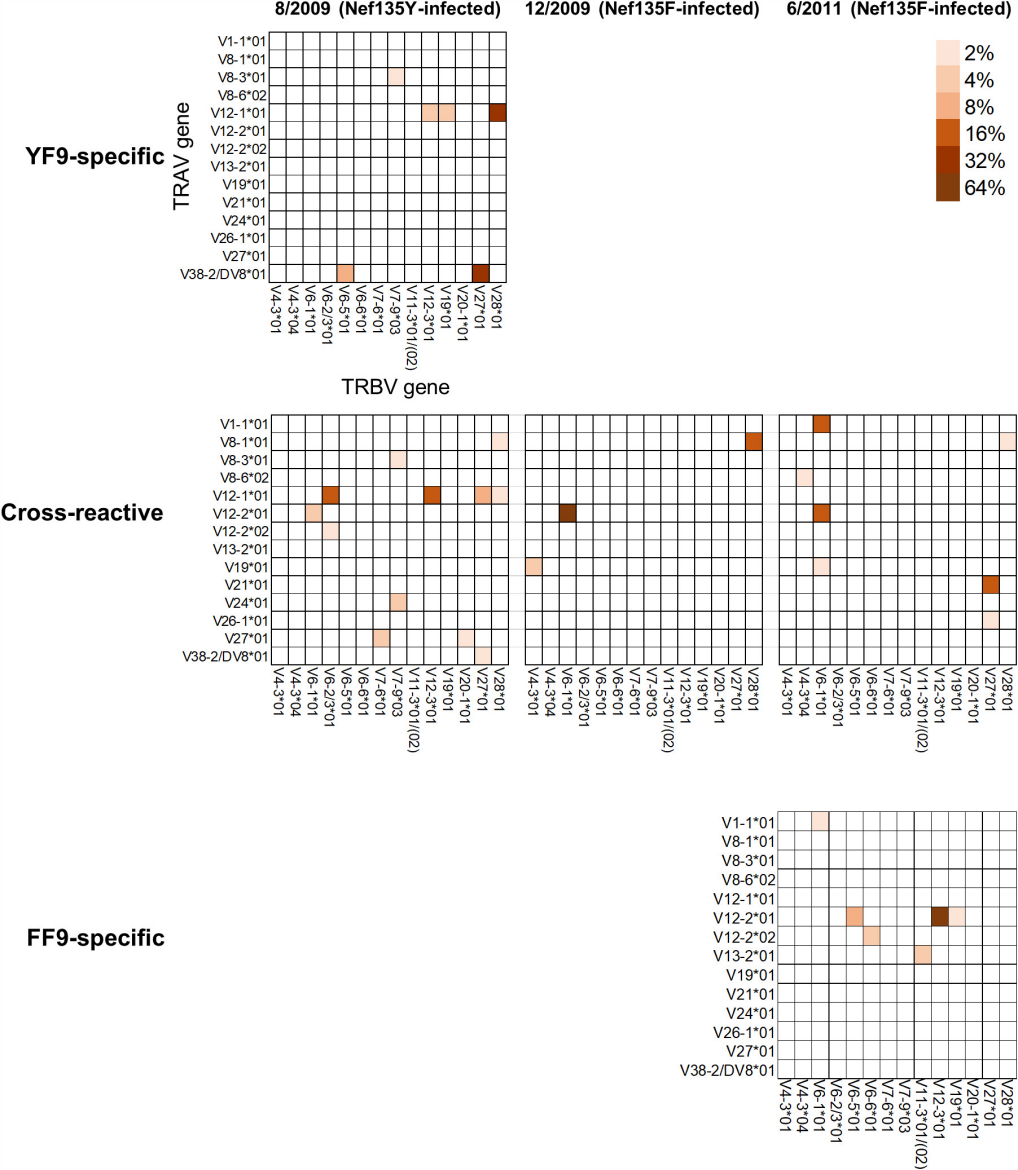
Longitudinal analysis of TCR clonotypes of YF9-specific, FF9-specific, or cross-reactive CD8^+^ T cells. Single YF9-specific, cross-reactive, and FF9-specific CD8^+^ T cells from KI-705 at early (August 2009 and December 2009) and chronic (June 2011) phases were sorted with a cell sorter after PBMCs from this individual had been stained with the YF9 and FF9 tetramers. TCR alpha and beta variable gene sequencing was identified at the single-cell level. Paired TCR α- and β-chain usage is shown. Thirty-one YF9-specific and 26 cross-reactive T cells in August 2009, 15 cross-reactive T cells in December 2009, and 16 FF9-specific T cells in June 2011 were analyzed.

Longitudinal T-cell analysis of KI-705 suggested that the emergence of the mutant virus induced a new T-cell repertoire able to recognize the FF9 mutant epitope. To clarify the dynamics of the specific CD8^+^ T-cell repertoire after the emergence of the Y135F mutation, we performed *ex vivo* TCR clonotype analysis of the HLA-B*35:01-restricted T cells at a single-cell level in this individual. We sorted the YF9-specific, the cross-reactive, and the FF9-specific CD8^+^ T cells using specific tetramers at 3 different time points and then analyzed the TCR clonotypes at the single-cell level (Fig. 3). When we analyzed the wild-type-specific and cross-reactive CD8^+^ T cells at the first time point (August 2009), 2 main TCR clonotypes, namely, TRAV12-1*01/TRBV28*01 and TRAV38-2/DV8*01/TRBV27*01, were found among the YF9-specific T cells, whereas 2 different main TCR clonotypes, namely, TRAV12-1*01/TRBV6-2/3*01 and TRAV12-1*01/TRBV12-3*01, were expressed predominantly on the cross-reactive T cells. TRAV12-1*01/TRBV28*01 and TRAV38-2/DV8*01/TRBV27*01 clonotypes were found in a small population of the cross-reactive T cells, whereas cells expressing TRAV12-1*01/TRBV12-3*01 were detected among the wild-type-specific T cells. This result might have been due to the contamination of one group by another during the cell sorting.

We next analyzed the cross-reactive CD8^+^ T cells at the second (December 2009) and third (June 2011) time points, which were during the phase after the mutant virus had emerged. Approximately 45% of YF9/FF9-specific T cells were cross-reactive T cells in December 2009. Only 3 TCR clonotypes were detected, although the dominant clonotypes were TRAV12-2*01/TRBV6-1*01 and TRAV8-1*01/TRBV28*01. These major clonotypes were found as a minor population in an earlier phase of infection when the wild-type virus predominated (August 2009). Three major TCR clonotypes, namely, TRAV1-1*01/TRBV6-1*01, TRAV12-2*01/TRBV6-1*01, and TRAV21*01/TRBV27*01, were found in June 2011, although the TRAV12-2*01/TRBV6-1*01 clonotype was also found in December 2009. Thus, the dominant TCR clonotypes among the cross-reactive T cells changed over a period of approximately 3 years.

We finally analyzed the mutant-specific CD8^+^ T cells that were a major population at the third time point (June 2011). Six TCR clonotypes were detected within the FF9-specific CD8^+^ T cells. Five of them were distinct from clonotypes found previously among the cross-reactive or wild-type-specific T cells, although one minor TCR clonotype, i.e., TRAV1-1*01/TRBV6-1*01, was also found within the cross-reactive T cells in June 2011. The T cells expressing this TCR clonotype among the mutant-specific T cells might have been contaminated by the cross-reactive T cells. Five TCR clonotypes were not detected either in the cross-reactive T cells or within the wild-type T cells, suggesting that they represented the mutant-specific TCR clonotypes in this individual. Overall, these findings indicate that the specific T cells with these 3 distinct specificities were elicited from different T-cell repertoires.

### Specificity of FF9-specific and cross-reactive CD8^+^ T-cell clones.

**FIG 4:**
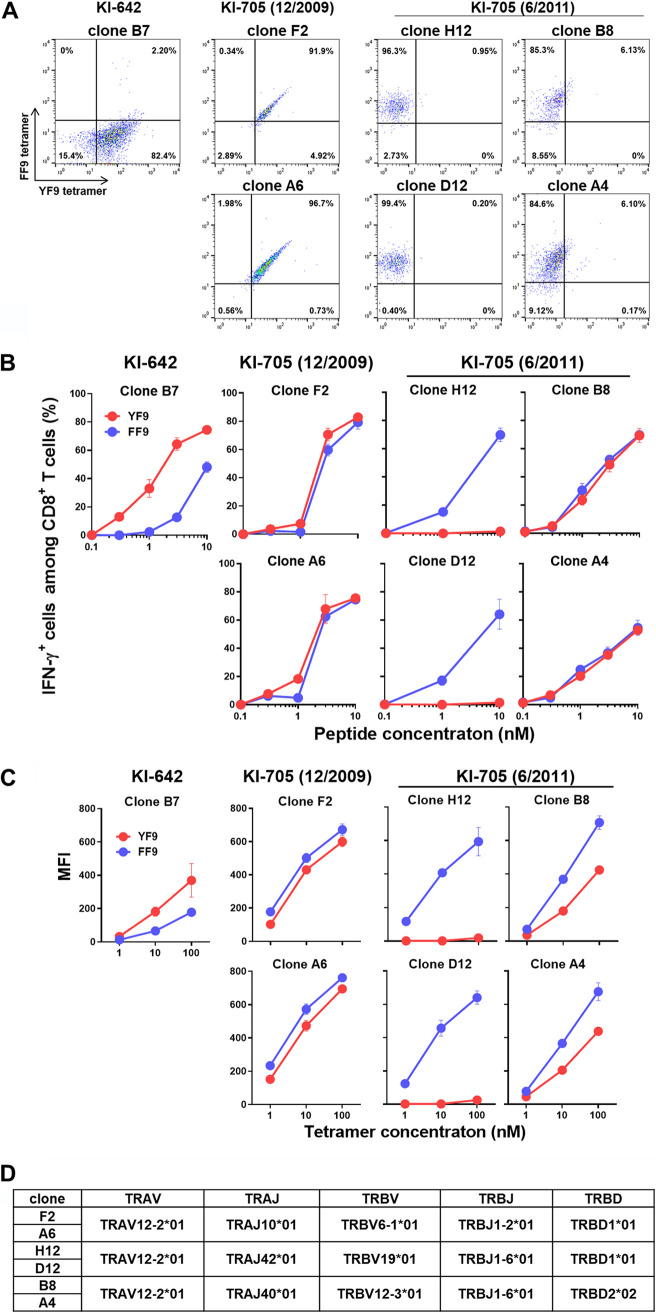
Phenotypic and functional analyses of 3 types of the HLA-B*35:01-restricted CD8^+^ T-cell clones. (A) Specificity of CD8^+^ T-cell clones. Six CTL clones (A6, F2, D12, H12, A4, and B8) and one clone (B7) were established from individuals KI-705 and KI-642, respectively. The specificity of these T-cell clones was determined by staining the clones with 100 nM HLA-B*35:01-YF9 and HLA-B*35:01-FF9 tetramers. (B) Recognition of YF9 or FF9 mutant peptides by the CTL clones. IFN-γ production from the CTL clones was detected by performing ICS assays. The data are presented as the mean and SD (*n* = 3). (C) Binding affinity of HLA-B*35:01-YF9 or HLA-B*35:01-FF9 tetramers to the T-cell clones. The binding of these tetramers to the clones was measured at 3 different concentrations (1, 10, and 100 nM). The data are presented as the mean and SD (*n* = 3). (D) TCR clonotype analysis of the 6 T-cell clones.

To characterize the specificity of the cross-reactive and FF9-specific T cells, we established T-cell clones with these specificities by sorting YF9-tet^+^FF9-tet^+^ and YF9-tet^−^FF9-tet^+^ CD8^+^ T cells from PBMCs from KI-705 in December 2009 and June 2011, respectively. Double staining analysis using the two tetramers demonstrated 2 cross-reactive (clones A6 and F2) and 4 FF9-specific T-cell clones (clones D12, H12, A4, and B8), while clones A4 and B8 showed weak binding affinity to the YF9-tetramer (Fig. 4A). We then analyzed the ability of these clones to recognize YF9 and FF9 peptides. Two cross-reactive clones showed similar levels of recognition for both the YF9 and FF9 peptides (Fig. 4B). Interestingly, FF9-specific T-cell clones showed 2 different recognition patterns for the YF9 and FF9 peptides. Two of them, namely, D12 and H12, recognized the FF9 mutant peptide but failed to respond to the YF9 peptide, whereas the other two, i.e., A4 and B8, showed similar levels of recognition for both the YF9 and FF9 peptides (Fig. 4B), suggesting that the A4 and B8 clones express a TCR which can recognize the wild-type peptide. Since we failed to establish YF9-specific T cells from this individual, we used as a control a YF9-specific cytotoxic T lymphocyte (CTL) clone (clone B7) derived from a wild-type virus-infected HLA-B*35:01^+^ individual, i.e., KI-642 (Fig. 4A). YF9-specific CTL clone B7 recognized the wild-type peptide more strongly than the FF9 variant (Fig. 4B).

To examine the TCR affinity of these T-cell clones, we investigated their binding affinities for the 2 HLA-B*35:01 tetramers. These T-cell clones were stained with YF9-tet and FF9-tet titrated from 100 nM to 1 nM. As expected, clone B7 showed a stronger binding affinity for YF9-tet than for FF9-tet (Fig. 4C, left), whereas the cross-reactive clones A6 and F2 showed similar binding affinities for both tetramers (Fig. 4C, middle). The FF9-specific clones D12 and H12 had a demonstrable binding affinity for FF9-tet but none at all for YF9-tet, whereas the mutant-specific clones A4 and B8 revealed strong binding to FF9-tet and relatively weak binding to YF9-tet (Fig. 4C, right), indicating that D12 and H12 T cells had FF9-specific TCRs, whereas A4 and B8 showed greater TCR affinity for the HLA-B*35:01-FF9 peptide complex than for the complex with YF9.

We next analyzed the TCR clonotype of these T-cell clones (Fig. 4D). The A6 and F2 cross-reactive clones expressed the major TCR clonotype of the cross-reactive T cells TRAV12-2*01/TRBV6-1*01. The D12 and H12 FF9-specific T-cell clones expressed a minor clonotype of FF9-specific T cells, namely, TRAV12-2*01/TRBV19*01, whereas the A4 and B8 FF9-specific T-cell clones showed a major clonotype among FF9-specific T cells, i.e., TRAV12-2*01/TRBV12-3*01. These findings indicate that the FF9-specific T cells, which had been classified by staining the T cells with the 2 HLA-B35:01 tetramers, could be divided into 2 groups, with one having FF9-specific TCRs and the other having TCRs with a significantly higher affinity for the FF9 epitope than for the wild-type peptide (YF9).

### HLA-B*35:01-restricted CD8^+^ T cells specific for the YF9/FF9 epitope were elicited in HLA-B*35:01^+^ individuals infected chronically with HIV-1.

**FIG 5:**
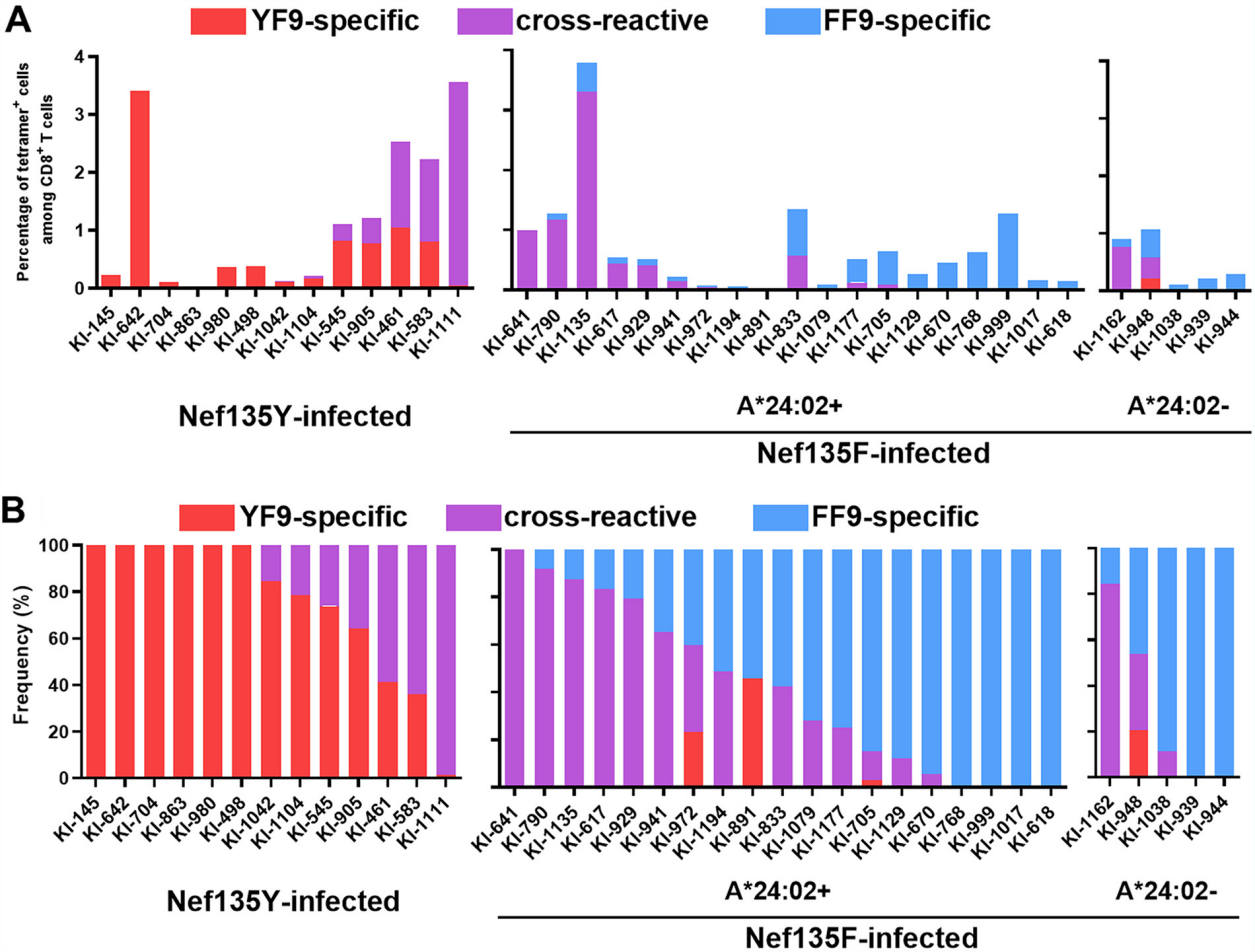
Cross-sectional analysis of HLA-B*35:01-restricted CD8^+^ T cells specific for the YF9 and/or FF9 epitope in HLA-B*35:01^+^ individuals infected chronically with the Nef135Y wild-type or Nef135F mutant viruses. Frequencies of YF9-specific, cross-reactive, and FF9-specific CD8^+^ T cells in 13 Nef135Y wild-type virus-infected and 24 Nef135F mutant virus-infected HLA-B*35:01^+^ individuals were determined by staining PBMCs *ex vivo* with both HLA-B*35:01-YF9 and HLA-B*35:01-FF9 tetramers. The percentage of HLA-B*35:01-YF9 tetramer^+^, HLA-B*35:01-FF9 tetramer^+^, and both tetramer^+^ CD8^+^ T cells among CD8^+^ T cells (A) and the frequency of these T cells among total tetramer^+^ CD8^+^ T cells (B) are presented for each individual.

The longitudinal analysis of patient KI-705 demonstrated the existence of HLA-B*35:01-restricted T cells exhibiting 3 patterns of specificities for YF9/FF9. We next investigated whether similar T-cell patterns could be elicited in HLA-B*35:01^+^ individuals infected either with the wild-type virus or with the Nef135F mutant virus at the population level. We analyzed PBMCs from 43 HIV-1 subtype B-infected HLA-B*35:01^+^ individuals by performing flow cytometry analysis using both YF9-tet and FF9-tet and found that 37 individuals had T cells that bound YF9-tet and/or FF9-tet (Fig. 5A), whereas the other 6 (1 infected with the wild-type virus and 5 with the mutant virus) did not (Table 1). Wild-type-specific and/or cross-reactive T cells were detected in 13 HLA-B*35:01^+^ individuals infected with the wild-type virus, whereas they showed no evidence of mutant-specific T cells (Fig. 5A and B). On the other hand, cross-reactive and/or mutant-specific T cells were detected in 24 individuals infected with the mutant virus, whereas a small population of wild-type-specific T cells were detected in some individuals (Fig. 5A). These wild-type-specific T cells, which had been elicited presumably during the phase of wild-type virus infection, may have been maintained as memory T cells in individuals infected with the mutant virus. These results support the idea that wild-type-specific and mutant-specific CD8^+^ T cells were elicited in HLA-B*35:01^+^ individuals infected with wild-type virus or mutant virus, respectively, whereas cross-reactive CD8^+^ T cells could be elicited in subjects infected with either the wild-type or mutant virus.

**TABLE 1:** Information on 43 HLA-B*35:01^+^ individuals chronically infected with HIV-1

Patient ID^*a*^	HLA-A alleles		HLA-B alleles		HLA-C alleles		Amino acid at Nef135	HLA-B*35:01-YF9/FF9-tetramer binding
KI-145	A*02:07	A*26:01	B*35:01	B*51:01	C*03:03	C*14:02	Y	+
KI-642	A*02:01	A*02:06	B*35:01	B*46:01	C*01:03	C*03:03	Y	+
KI-704	A*11:01	A*31:01	B*15:11	B*35:01	C*03:03	C*03:03	Y	+
KI-863	A*11:01	A*33:03	B*35:01	B*44:03	C*01:23	C*14:03	Y	+
KI-980	A*02:06	A*11:01	B*35:01	B*40:01	C*04:01	C*07:02	Y	+
KI-498	A*02:06	A*31:01	B*35:01	B*40:02	C*03:03	C*03:04	Y	+
KI-1042	A*02:06	A*24:02	B*35:01	B*55:02	C*03:03	C*03:04	Y	+
KI-1104	A*24:02	A*31:01	B*35:01	B*40:06	C*03:03	C*03:04	Y	+
KI-545	A*02:06	A*26:03	B*15:01	B*35:01	C*03:03	C*03:04	Y	+
KI-905	A*26:01	A*33:03	B*35:01	B*44:03	C*03:03	C*14:03	Y	+
KI-461	A*26:01	A*31:01	B*35:01	B*51:01	C*03:03	C*14:02	Y	+
KI-583	A*02:01	A*02:01	B*35:01	B*48:01	C*08:01	C*08:01	Y	+
KI-1111	A*11:01	A*26:01	B*35:01	B*52:01	C*03:03	C*12:02	Y	+
KI-1056	A*24:02	A*24:02	B*35:01	B*35:01	C*08:01	C*08:01	Y	−
KI-641	A*24:02	A*31:01	B*35:01	B*51:01	C*03:03	C*14:02	F	+
KI-790	A*24:02	A*31:01	B*15:01	B*35:01	C*03:03	C*04:01	F	+
KI-1135	A*24:02	A*24:02	B*35:01	B*52:01	C*03:03	C*12:02	F	+
KI-617	A*02:06	A*24:02	B*35:01	B*40:01	C*03:03	C*03:04	F	+
KI-929	A*24:02	A*26:03	B*35:01	B*52:01	C*03:03	C*12:02	F	+
KI-941	A*02:06	A*24:02	B*35:01	B*40:02	C*03:04	C*03:04	F	+
KI-972	A*24:02	A*26:01	B*35:01	B*52:01	C*03:03	C*12:02	F	+
KI-1194	A*24:02	A*26:01	B*35:01	B*40:01	C*03:03	C*03:04	F	+
KI-891	A*02:06	A*24:02	B*35:01	B*59:01	C*01:02	C*03:03	F	+
KI-833	A*24:02	A*31:01	B*35:01	B*48:01	C*08:01	C*08:01	F	+
KI-1079	A*02:06	A*24:02	B*35:01	B*52:01	C*03:03	C*12:02	F	+
KI-1177	A*11:01	A*24:02	B*35:01	B*51:01	C*08:01	C*15:02	F	+
KI-705	A*24:02	A*26:01	B*35:01	B*52:01	C*03:03	C*12:02	F	+
KI-1129	A*24:02	A*24:02	B*35:01	B*52:01	C*08:01	C*1202	F	+
KI-670	A*02:06	A*24:02	B*15:01	B*35:01	C*03:03	C*04:01	F	+
KI-768	A*02:07	A*24:02	B*07:02	B*35:01	C*03:03	C*07:02	F	+
KI-999	A*24:02	A*24:02	B*15:01	B*35:01	C*03:03	C*04:01	F	+
KI-1017	A*02:06	A*24:02	B*35:01	B*54:01	C*01:02	C*03:03	F	+
KI-618	A*24:02	A*24:02	B*35:01	B*67:01	C*07:02	C*08:01	F	+
KI-1162	A*02:01	A*31:01	B*07:02	B*35:01	C*07:02	C*08:03	F	+
KI-948	A*02:06	A*02:06	B*35:01	B*35:01	C*0303	C*03:03	F	+
KI-1038	A*02:01	A*02:01	B*15:01	B*35:01	C*03:03	C*0801	F	+
KI-939	A*02:01	A*26:01	B*35:01	B*54:01	C*01:02	C*03:03	F	+
KI-944	A*02:06	A*26:01	B*35:01	B*40:02	C*03:03	C*03:23	F	+
KI-605	A*24:02	A*24:20	B*35:01	B*46:01	C*01:02	C*03:03	F	−
KI-856	A*02:01	A*31:01	B*54:01	B*35:01	C*01:02	C*08:01	F	−
KI-1022	A*24:28	A*33:03	B*35:01	B*44:03	C*03:03	C*15:02	F	−
KI-1028	A*11:01	A*24:02	B*35:01	B*51:01	C*08:01	C*15:02	F	−
KI-1084	A*02:06	A*24:02	B*35:01	B*59:01	C*01:02	C*03:03	F	−

a ID, identifier.

We speculated that in some cases the Nef135F mutant virus was transmitted from HLA-A*24:02^+^ individuals to HLA-A*24:02^−^ subjects, since this mutation is selected by HLA-A*24:02-restricted T cells. Therefore, we expected that HLA-A*24:02^−^HLA-B*35:01^+^ individuals with the mutant virus would have cross-reactive and mutant-specific T cells but not wild-type-specific T cells. Four of 5 HLA-A*24:02^−^HLA-B*35:01^+^ individuals infected with the mutant virus did not have wild-type-specific T cells, whereas patient KI-948 had wild-type-specific T cells along with cross-reactive and mutant-specific T cells (Fig. 5A). We speculate that a mixture of viruses was transmitted to KI-948 and then wild-type virus-specific T cells were elicited by wild-type virus-infected cells.

### Potential of the three different specificities of HLA-B*35:01-restricted YF9/FF9-specific CD8^+^ T cells to suppress HIV-1 replication in HIV-1-infected HLA-B*35:01^+^ individuals.

**FIG 6:**
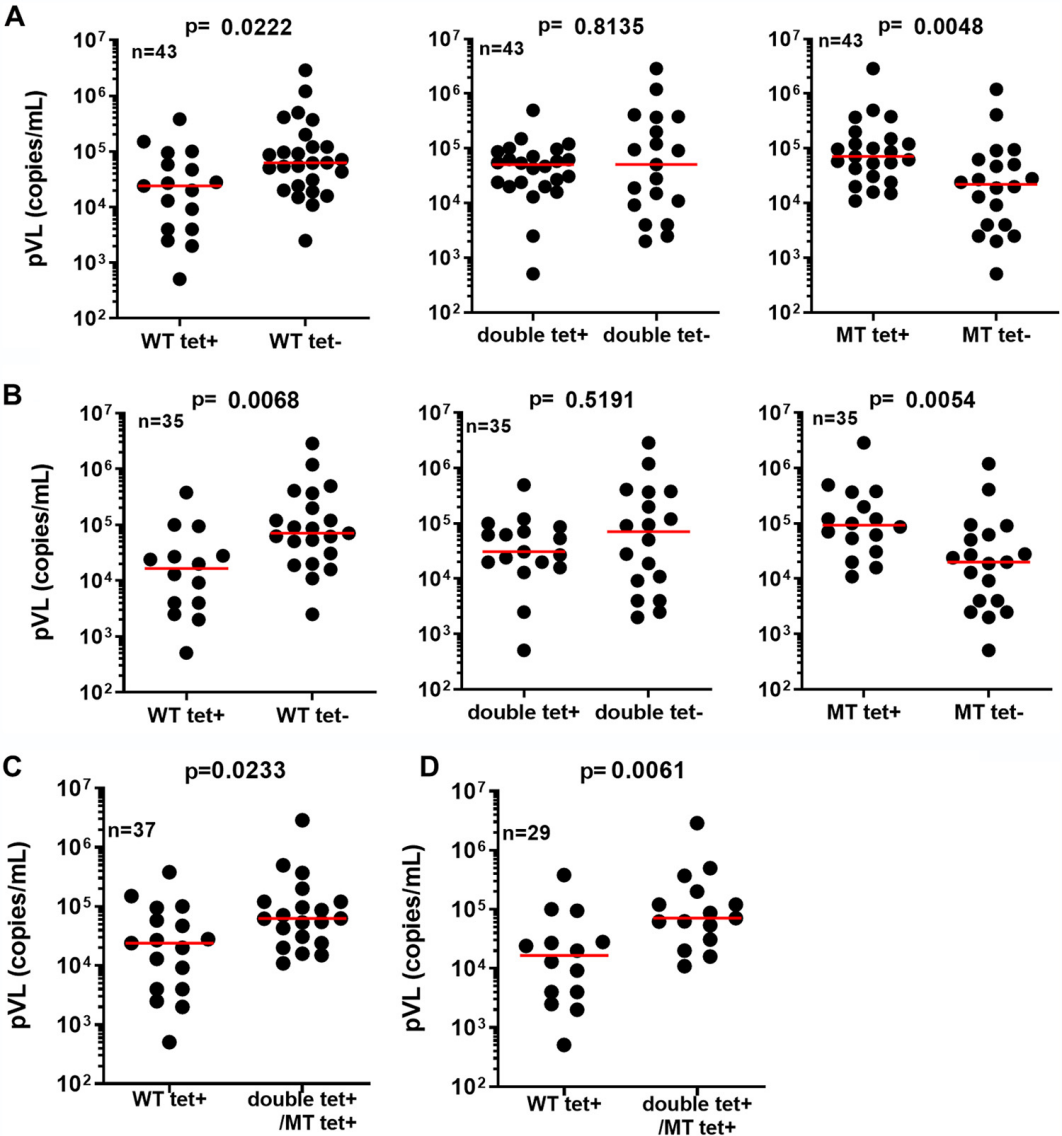
Role of HLA-B*35:01-restricted YF9/FF9-specific CD8^+^ T cells in the suppression of HIV-1 replication *in vivo.* pVL values were compared between HIV-1-infected individuals who had CD8^+^ T cells bound to HLA-B35:01-YF9 tetramer (WT tet^+^) and those who did not (WT tet^−^), between HLA-B*35:01^+^ individuals who had CD8^+^ T cells bound to both the HLA-B*35:01-YF9 and HLA-B*35:01-FF9 tetramers (double tet^+^) and those who did not (double tet^−^), and between HLA-B*35:01^+^ individuals who had CD8^+^ T cells bound to HLA-B*35:01-FF9 tetramer (MT tet^+^) and those who did not (MT tet^−^) for 43 HLA-B*35:01^+^ individuals (A) and for 35 HLA-B*35:01^+^ individuals who had neither the protective haplotype HLA-B*52:01-C*12:02 nor protective allele HLA-B*67:01 (B). pVLs were compared among HIV-1-infected individuals who had CD8^+^ T cells binding the HLA-B*35:01-YF9 tetramer (WT tet^+^) those who had CD8^+^ T cells bound to both HLA-B*35:01-YF9 and HLA-B35:01-FF9 tetramers or to the HLA-B*35:01-FF9 tetramer (double-tet^+^/MT tet^+^) for 37 HLA-B*35:01^+^ individuals (C) and for 29 HLA-B*35:01^+^ individuals who had neither HLA-B*52:01-C*12:02 nor HLA-B*67:01 (D). Statistical analyses were performed using the Mann-Whitney test. MT, mutant.

To investigate the role of HLA-B*35:01-restricted CD8^+^ T cells with each of the three different specificities in the suppression of HIV-1 replication *in vivo*, we analyzed the correlation between the presence of T cells with each specificity and plasma viral load (pVL) in 43 HIV-1-infected HLA-B*35:01^+^ individuals. Details of these individuals are shown in Table 1. Individuals possessing wild-type-specific T cells had a significantly lower pVL than those without them, whereas patients with mutant-specific T cells had a significantly higher pVL than those without that specificity (Fig. 6A). On the other hand, no significant difference was found between individuals with or without cross-reactive T cells (Fig. 6A). These results suggest that the wild-type-specific T cells maintained the ability to suppress HIV-1 replication *in vivo*, whereas mutant-specific and cross-reactive T cells did not. HLA-B*52:01, HLA-C*12:02, and HLA-B*67:01 are protective alleles in Japan (30). We next analyzed the remaining 35 HIV-1-infected HLA-B*35:01^+^ individuals after excluding those who had either the HLA-B*52:01-C*12:02 haplotype or the HLA-B*67:01 allele in order to exclude the effect of these protective alleles. There was still a negative association between the presence of WT-specific T cells and pVL and a positive association between the detection of mutant-specific T cells and pVL (Fig. 6B). An analysis of the frequency of these 3 types of T cells and clinical outcome also showed a positive correlation and confirmed the likely role of wild-type-specific T cells in good clinical outcome (data not shown).

Out of 43 individuals analyzed, 6 individuals did not have HLA-B3501-tetramer^+^ T cells. We next divided 37 individuals possessing any T cells specific for the YF9 or FF9 epitope into 2 groups, i.e., individuals with wild-type-specific T cells and those without them but possessing cross-reactive or mutant-specific T cells. We found that individuals with wild-type-specific T cells had a significantly lower pVL than those who lacked them but did have cross-reactive or mutant-specific T cells (Fig. 6C). We further compared the pVL in these 2 groups after excluding the 8 individuals who had protective alleles. We found very similar results in both the larger group and with the exclusion of those with protective alleles (Fig. 6D). These results together indicate that wild-type-specific T cells had the ability to suppress HIV-1 replication *in vivo*, whereas the mutant-specific and cross-reactive T cells did not.

One can speculate that HLA-A*24:02-restricted RF10-specific T cells may have contributed to the suppression of HIV-1 replication in HLA-A*24:02^+^HLA-B*35:01^+^ individuals. A previous study showed no significant difference in pVL between responders to the RF10 or RF10-2F mutant epitope and nonresponders in mutant virus-infected HLA-A*24:02^+^HLA-B*35:01^+^ individuals (34), suggesting that these HLA-A*24:02-restricted T cells do not directly suppress HIV-1 replication *in vivo* but would have had an indirect impact on pVL in these individuals via the selection of the Nef135F mutant virus.

### Recognition of HIV-1-infected cells by 3 types of HLA-B*35:01-restricted YF9/FF9-specific T-cell lines.

**FIG 7:**
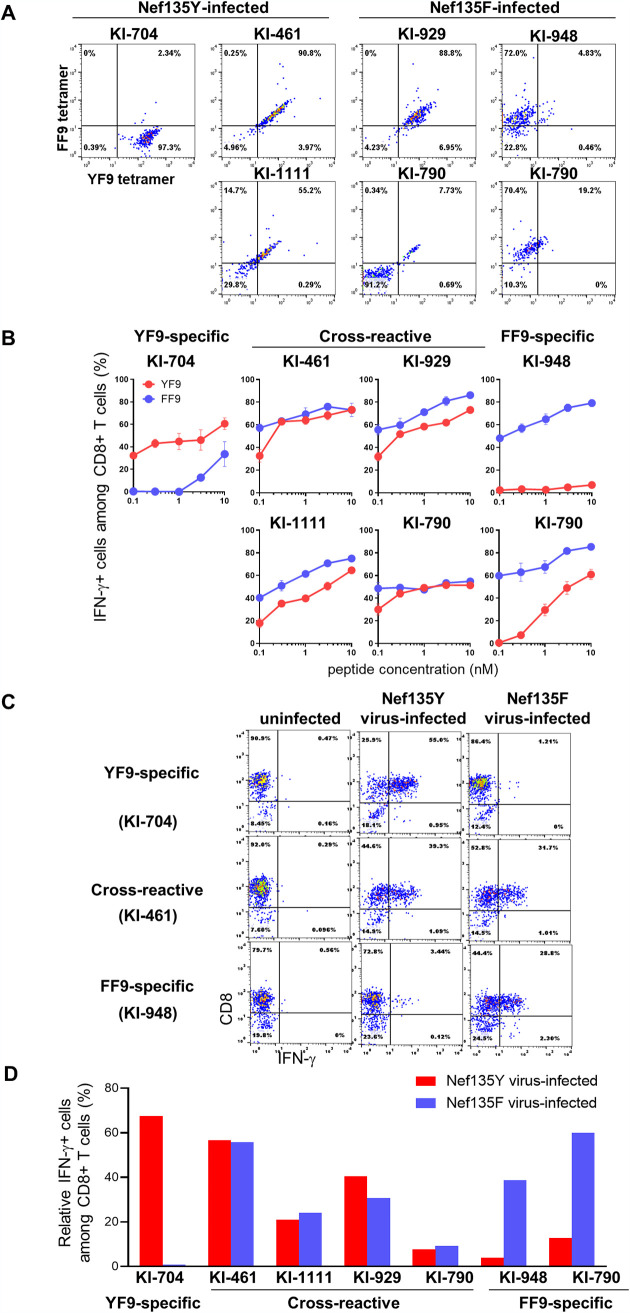
Recognition of HIV-1-infected cells by 3 types of YF9/FF9-specific HLA-B*35:01-restricted CD8^+^ T-cell lines. Three types of YF9/FF9-specific HLA-B*35:01-restricted CD8^+^ T-cell lines were established from HLA-B*35:01^+^ individuals infected with the wild-type virus (KI-704, KI-461, and KI-1111) or those with the mutant virus (KI-790, KI-929, and KI-948). The specificity of these T-cell lines was determined by staining these T-cell lines with 100 nM HLA-B*35:01-YF9 and HLA-B*35:01-FF9 tetramers (A) and by performing the ICS assay for the recognition of peptide-pulsed target T cells (B). Recognition by these T-cell lines of 721.221-B*35:01 cells infected with the NL4-3-Nef135Y (Nef135Y) or those with NL4-3-Nef135F (Nef135F) virus was analyzed using the ICS assay (C and D). The frequencies of p24 antigen-positive cells among 721.221-B*35:01 cells infected with the Nef135Y virus and those with the Nef135F strain were 17.1% and 18.1%, respectively.

We next analyzed how well these three T-cell populations with distinct specificities could recognize CD4^+^ T cells infected either with the Nef135 mutant virus or with the wild-type virus. T cells with these specificities were established from 3 HLA-B*35:01^+^ individuals infected with the wild-type virus (KI-704, KI-461, and KI-1111) or 3 with a mutant virus (KI-790, KI-929, and KI-948). We stained PBMCs with YF9-tet and FF9-tet and then sorted the tetramer^+^ CD8^+^ T cells. The sorted T cells derived from KI-704, KI-461, and KI-1111 and those from KI-790, KI-929, and KI-948 were stimulated with the YF9 and the FF9 peptide, respectively, and then they were cultured for approximately 2 weeks. Wild-type-specific and cross-reactive T-cell lines were generated from KI-704, KI-461, and KI-1111; whereas mutant-specific and cross-reactive T cells were elicited from KI-790, KI-929, and KI-948. We analyzed the specificity of these T-cell lines by staining them with both tetramers (Fig. 7A) and by performing intracellular cytokine staining (ICS) assays for the recognition of peptide-pulsed target cells (Fig. 7B). In this way, 1 wild-type-specific, 4 cross-reactive, and 2 mutant-specific T-cell lines were established.

We next investigated whether these T-cell lines could recognize target T cells infected with either the wild-type virus or the mutant virus (Fig. 7C). The wild-type-specific T-cell line recognized target cells infected with the wild-type (Nef135Y) virus but not those infected with the mutant (Nef135F) strain, whereas 2 mutant-specific T cells recognized target cells infected with the Nef135F virus but only weakly recognized those infected with the Nef135Y virus (Fig. 7D). Four cross-reactive T cells effectively recognized target cells infected with either virus (Fig. 7D). These results demonstrated that these T cells could effectively recognize wild-type virus-infected and mutant virus-infected cells based on their epitope specificity.

### Expression of molecules associated with T-cell function in HLA-B*35:01-restricted YF9/FF9-specific T-cell lines with different specificities.

**FIG 8:**
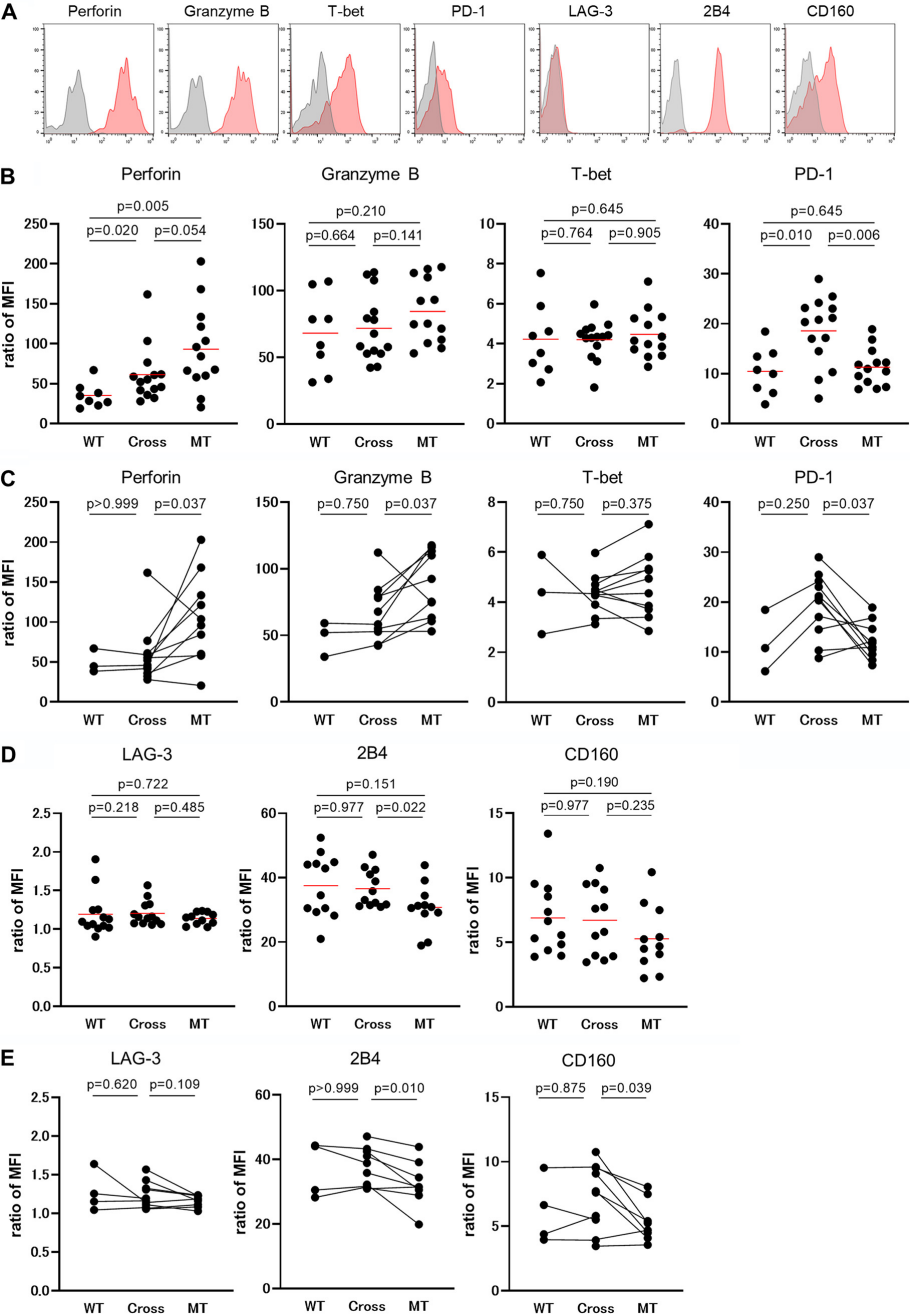
Expression of inhibitory molecules and molecules associated with T-cell function in 3 types of HLA-B*35:01-restricted YF9/FF9-specific T cells. PBMCs from 22 HLA-B*35:01^+^ individuals who had HLA-B*35:01-restricted YF9/FF9-specific T cells were stained with both YF9-tet and FF9-tet as well as with MAbs specific for PD-1, perforin, granzyme B, or T-bet; while PBMCs from 23 HLA-B*35:01^+^ individuals who had HLA-B*35:01-restricted YF9/FF9-specific T cells were stained with both YF9-tet and FF9-tet as well as with MAbs specific for inhibitory molecule LAG-3, 2B4, or CD160. (A) Representative staining results of each molecule (red) or the corresponding isotype control (gray) of YF9/FF9-specific T cells. (B) Summarized results of the relative expression levels of PD-1, perforin, granzyme B, and T-bet in 3 types of T cells from 22 HLA-B*35:01^+^ individuals. Seven and 15 individuals were infected with wild-type and mutant viruses, respectively. (C) Relative expression levels of PD-1, perforin, granzyme B, and T-bet in 3 types of T cells from the same individuals. (D) Summarized results of the relative expression levels of LAG-3, 2B4, and CD160 in 3 types of T cells from 23 HLA-B*35:01^+^ individuals. (E) Relative expression levels of LAG-3, 2B4, and CD160 in 3 types of T cells from the same individuals. The expression level of perforin, granzyme B, T-bet, or inhibitory/stimulatory molecules in tetramer^+^ CD8^+^ T cells from each individual was compared in terms of the ratio of MFI. The ratio of MFI was calculated as the MFI of molecule/that of isotype control. Statistical analyses were performed using the Mann-Whitney test (B, D) or Wilcoxon signed-rank test (C, E).

We found different functional abilities of HLA-B*35:01-restricted FF9-specific and cross-reactive T cells between *in vitro* and *in vivo* situations. We therefore investigated the expression of molecules associated with T-cell function, such as PD-1, perforin, granzyme B, and T-bet, in the 3 types of *ex vivo* HLA-B*35:01-restricted YF9/FF9-specific T cells. We analyzed PBMCs from 22 HLA-B*35:01^+^ individuals who had HLA-B*35:01-restricted YF9/FF9-specific T cells. The analysis was done using both YF9-tet and FF9-tet, as well as monoclonal antibodies (MAbs) specific for PD-1, perforin, granzyme B, or T-bet. The results from analyses of these 22 individuals showed that both mutant-specific and cross-reactive T cells showed a significantly higher expression of perforin than wild-type-specific T cells and that cross-reactive T cells expressed a significantly higher level of PD-1 than either the wild-type-specific or mutant-specific T cells (Fig. 8A). Mutant-specific T cells also showed a significantly higher expression of perforin and lower expression of PD-1 than the cross-reactive T cells in the same individuals (Fig. 8B). No significant difference in the expression of granzyme or T-bet was found among these 3 T-cell populations. These findings indicate that a higher expression of PD-1 may be linked with a reduced ability of cross-reactive T cells to suppress HIV-1 replication *in vivo.*

Since PD-1 expression could not explain the lower ability of mutant-specific T cells to suppress HIV-1 replication *in vivo*, we further analyzed the expression of 3 molecules (2B4, LAG-3, and CD160) associated with T-cell function on these T cells. Mutant-specific T cells showed a trend to express CD160 and 2B4 at lower levels than wild-type-specific or cross-reactive T cells (Fig. 8C). When we compared the expression of CD160 and 2B4 between cross-reactive and mutant-specific T cells in the same individuals (*n* = 8), the mutant-specific T cells expressed these molecules at significantly lower levels than cross-reactive T cells (Fig. 8D). On the other hand, no significant difference was found between wild-type-specific and cross-reactive T cells in the same individuals (*n* = 4). It is known that CD160 and 2B4 have both inhibitory and stimulatory roles with respect to effector functions in T cells (35–37) and that HIV-1-specific CD8^+^ T cells in elite controllers or slow progressors express higher CD160 levels than chronic progressors (38, 39). These findings suggest that the lower expression of CD160 in the mutant-specific T cells may account for their reduced function *in vivo*.

## DISCUSSION

Previous studies of HLA-associated HIV-1 polymorphisms in Japan showed that the NefY135F mutation is associated with HLA-A*24:02 but not with HLA-B*35:01 (15), as this mutation is selected by HLA-A*24:02-restricted NefRF10-specific and/or Nef RW8-specific T cells (11, 21). These findings strongly suggest that the NefY135F mutation is not selected by HLA-B*35:01-restricted T cells. In the present study, we showed that HLA-B*35:01-restricted YF9-specific CD8^+^ T cells could recognize target cells infected with the Nef135Y wild-type virus but failed to recognize those infected with the NefY135F mutant virus. This result suggests that the mutant virus could be selected by YF9-specific T cells in HIV-1-infected HLA-B*35:01^+^ individuals. However, in a longitudinal analysis of these T cells in individual KI-705 who had both HLA-A*24:02 and HLA-B*35:01, we demonstrated that HLA-A*24:02-restricted NefRF10-specific T cells were elicited in the early phase of the wild-type virus infection but that HLA-B*35:01-restricted NefYF9-specific T cells were not (Fig. 2C). Thus, since HLA-A*24:02-restricted T cells were elicited much earlier than the HLA-B*35:01-restricted T cells, it is likely that the HLA-A*24:02-restricted T cells, not the HLA-B*35:01-restricted T cells, were responsible for selecting the Nef135F mutation. Among 13 wild-type virus-infected HLA-B*35:01^+^ individuals who had wild-type-specific and/or cross-reactive T cells, only 2 individuals (15%) also expressed HLA-A*24:02 (Table 1). In contrast, among 23 mutant virus-infected HLA-B*35:01^+^ individuals who had mutant-specific and/or cross-reactive T cells, 18 of them (78%) also had HLA-A*24:02 (Fig. 5 and Table 1). These findings also support the idea that the Nef135F mutation was selected by HLA-A*24:02-restricted T cells rather than by HLA-B*35:01-restricted responses.

In the analysis of HLA-B*35:01-restricted CD8^+^ T cells specific for the YF9/FF9 epitope, performed using 2 HLA-B*35:01 tetramers to stain PBMCs from 43 HIV-1-infected HLA-B*35:01^+^ Japanese individuals, we found that HLA-B*35:01-restricted CD8^+^ T cells specific for the YF9/FF9 epitope were elicited in 37 of them. We further showed that FF9-specific T cells were elicited in 23 (95.8%) of 24 HLA-B*35:01^+^ individuals infected with the Nef135F mutant virus. FF9-mutant specific T cells were elicited in most of the individuals infected with the Nef135F mutant virus, and they effectively recognized target cells infected with the mutant virus. These findings indicate that the FF9 mutant peptide was presented effectively in mutant virus-infected cells. A previous study showed that the affinity of the FF9 peptide for HLA-B*35:01 is almost identical to that of the YF9 wild-type peptide (34). This finding could account for the effective presentation of the FF9 mutant epitope and elicitation of FF9-specific and cross-reactive T cells in individuals infected with the Nef135F mutant virus.

The longitudinal analysis of patient KI-705 demonstrated that both wild-type-specific and cross-reactive CD8^+^ T cells were elicited during the phase of wild-type virus infection. RNA deep sequencing analysis demonstrated that the nucleotide at Nef135 in this phase was almost entirely the wild-type one (A at all of the 89 reads at nucleotide position 9200), supporting the idea that the cross-reactive T cells could be elicited in response to the wild-type virus. The cross-sectional analysis of 13 HLA-B*35:01^+^ individuals infected with the wild-type virus also showed that both the wild-type-specific and cross-reactive T cells could be detected in 7 of these individuals (Fig. 5). A previous study demonstrated that cross-reactive T cells recognizing both the HLA-A*24:02-restricted epitope GagKW9 and its mutant epitope GagKW9-3R are elicited in HLA-A*24:02^+^ individuals infected with the wild-type virus (22). These findings together suggest that cross-reactive T cells could be elicited during wild-type virus infection. Cross-reactive T cells were also found during a phase of mutant virus infection in patient KI-705 and in 23 of 24 HLA-B*35:01^+^ individuals infected with the mutant virus (Fig. 5). The role of these cross-reactive T cells in HIV-1 infection is unclear; one possible role may be to prevent the accumulation of the mutant virus in a phase of wild-type virus infection and/or reversion to wild-type virus in a phase of mutant virus infection.

Longitudinal TCR clonotype analysis of KI-705 demonstrated that the TCR clonotypes of wild-type-specific T cells were different from those of mutant-specific T cells and that the major TCR clonotypes of cross-reactive T cells were also distinct from those of wild-type-specific and mutant-specific T cells. Thus, the present study demonstrated that different TCR clonotypes were found among T cells with these 3 different specificities. This finding suggests that these 3 types of T cells were derived from different naive T-cell repertoires. This suggestion is consistent with the results from a previous study of TCR clonotype analysis of RF10/RF10-2F-specific HLA-A*24:02-restricted T cells, which showed that TCR clonotypes were different between RF10-specific and RF10-2F mutant-specific T cells in 3 HIV-1 infected individuals (11). Further studies of TCR clonotypes with different HIV-1 antigen specificities performed with longitudinal analysis within the same individuals will be able to clarify the mechanism by which mutant epitope-specific T cells are induced in HIV-1 infection.

Two main TCR clonotypes (TRAV12-1*01/TRBV6-2/3*01 and TRAV12-1*01/TRBV12-3*01) were predominant among cross-reactive T cells during the initial phase of wild-type virus infection but not in the subsequent early and chronic phases of mutant virus infection. Six of 8 minor clonotypes found during wild-type virus infection were not detected in either the early or chronic phases of mutant virus infection, whereas TRAV12-2*01/TRBV6-1*01 and/or TRAV8-1*01/TRBV28*01 clonotypes were found as a major population of cross-reactive T cells in early and chronic phases of mutant virus infection. These findings suggest that only a small population of the cross-reactive T-cell population that are generated during wild-type virus infection could expand after the emergence of the mutant virus. TRAV1-1*01/TRBV6-1*01 and TRAV21*01/TRBV27*01 were found as major clonotypes only in the chronic phase of mutant virus infection. The cross-reactive T-cell clones with these TCR clonotypes may have been selected and elicited more effectively by mutant virus-infected cells than by wild-type virus-infected cells. Thus, cross-reactive T cells with distinct TCR repertoires were elicited after the emergence of the mutant virus.

An analysis of TCR affinity and the clonotype of FF9-specific T-cell clones derived from individual KI-705 showed that T cells expressing a minor clonotype, TRAV12-2*01/TRBV19*01, had TCRs specific for the FF9 mutant epitope (FF9-specific TCR); whereas T-cell clones expressing a major clonotype, TRAV12-2*01/TRBV12-3*01, had TCRs with higher affinity for the HLA-B*35:01-FF9 peptide complex than for the HLA-B*35:01-YF9 combination (FF9-dominant TCR). Thus, FF9-specific T cells expressing TCRs with 2 different specificities for the FF9/YF9 epitope were elicited in the chronic phase. T cells expressing FF9-specific TCR and those expressing FF9-dominant TCR were also elicited in patients KI-948 and KI-790, respectively. Both types of FF9-specific T cells showed potent ability to recognize target cells infected with the mutant virus, whereas only T cells expressing FF9-dominant TCR could recognize target cells infected with wild-type virus. The role of T cells expressing FF9-dominant TCR in HIV-1-infected individuals is unknown, but we speculate that these T cells may prevent the reversion to wild-type virus.

A previous study showed that Nef135Y/135L wild-type virus-infected responders to the YF9 peptide have a much lower pVL than Nef135F mutant virus-infected responders to the YF9 peptide (34), suggesting that the emergence of the Y135F mutant impairs the ability of YF9-specific CD8^+^ T cells to suppress the replication of the mutant virus in the HLA-B*35:01^+^ individuals. The present study further demonstrated that HLA-B*35:01^+^ individuals with wild-type-specific T cells, who were infected mostly with the wild-type virus, had a significantly lower pVL than those who lacked wild-type-specific T cells but had mutant-specific and/or cross-reactive T cells. In contrast, individuals with FF9 mutant-specific T cells who had been infected with the Nef135F mutant virus had a significantly higher pVL than those with wild-type-specific and/or cross-reactive T cells. These findings suggest that mutant-specific and cross-reactive T cells were not able to suppress the replication of HIV-1 mutant virus *in vivo*, even though these T cells could be elicited during mutant virus infection and could recognize mutant virus-infected cells. Although we did not measure viral reservoir size in the present study, it might be expected from these findings that T cells generated later in HIV infection against emerging escape mutants have a reduced capacity to control the viral reservoir and would be poor candidates for immune-based therapy in viral eradication strategies.

It is well known that PD-1 expression correlates with the impairment of CD8 T-cell functionality, increased viral load, and reduced CD4 T-cell counts in HIV-1 infection (40–46). We therefore speculated that cross-reactive or mutant-specific T cells would express an elevated level of PD-1 *in vivo*. Indeed, the discrepancy in function of cross-reactive T cells between the *in vitro* and *in vivo* situations could be explained by the higher PD-1 expression on cross-reactive T cells *in vivo* (Fig. 8A and B). On the other hand, mutant-specific T -cells expressed lower levels of CD160 and 2B4 than cross-reactive T cells when we compared the expression levels in the same individuals (Fig. 8D). Since these molecules are known to have the capacity both to stimulate and inhibit cytolytic activity (35–39), the lower expression of these molecules might affect the function of the mutant-specific T cells *in vivo*. A previous study showed that elite controllers expressed CD160 at higher levels than chronic progressors and demonstrated that they harbor a population of HIV-specific CD160^+^2B4^+^CD8^+^ T cells that correlates with cytolytic capacity (38). A recent study also demonstrated that the frequency of CD160^+^CD8^+^ T cells was higher in slow progressors than that in progressors and that CD160^+^CD8^+^ T cells showed stronger activity in responding to HIV-1 Gag peptides than CD160^−^CD8^+^ T cells (39). These studies suggested that CD160 may play a protective role in suppressing HIV-1 replication. From these findings, we speculate that the negative signal via PD-1 is much stronger than the positive signal via CD160/2B4 in cross-reactive T cells, while the lack of a positive signal via CD160/2B4 critically affects the function of mutant-specific T cells. However, no direct evidence is provided in the present study. Other mechanisms, as yet unknown, might account for the poor function of the mutant-specific T cells against HIV-1 replication. Further studies are needed to clarify the underlying mechanisms.

We demonstrated the effect of the HLA-A*24:02-associated Nef135F mutation on the generation of FF9-specific and cross-reactive T cells and the association of the emergence of these populations with clinical outcome. Three types of HLA-B*35:01-restricted T cells specific for YF9/FF9 were elicited during the clinical course, starting from a phase of wild-type virus infection to that dominated by mutant virus. Individuals with YF9-specific T cells had a significantly lower pVL than those with FF9-specific and/or cross-reactive T cells, whereas those with FF9 mutant-specific T cells had a significantly higher pVL than those with YF9-specific and/or cross-reactive T cells. Thus, although the Nef135F escape mutant selected by HLA-A*24:02-restricted T cells could elicit mutant-specific and cross-reactive HLA-B*35:01-restricted T cells, these T cells could not effectively suppress HIV-1 replication *in vivo*, even though they could recognize mutant virus-infected cells *in vitro*. This finding might be explained by the higher expression of PD-1 on cross-reactive T cells and lower expression of CD160/2B4 on the mutant-specific T cells. Thus, here, we demonstrated the collaboration of two HLA class I alleles in the coevolution of HIV-1 and HIV-1-specific T cells, as well their contribution to the capacity of HLA-B*35:01-restricted T cells to affect the clinical outcome. Compared with the accumulation of the NefY135F mutation in Japanese individuals (64%, 84%, and 26% of total, HLA-A*24:02^+^, and HLA-A*24:02^−^ individuals, respectively) (15), the Nef135 mutation was found at a similar level (approximately 70%) in HLA-A*24:02^+^ individuals but in approximately 10% and 20% of HLA-A*24:02^−^ and total individuals among Caucasian and U.S. cohorts (47). This information suggests that FF9-specific T cells may be elicited in some HLA-B*35:01^+^ individuals infected with the NefY135F mutant virus in these cohorts. The accumulation of this mutant and the induction of FF9-specific T cells in HIV-1-infected HLA-B*35:01^+^ individuals are also expected in countries such as Mexico, South Korea, and Italy where both HLA-A*24:02 and -B*35:01 are detected frequently. The analysis of the NefY135F mutation and of the mutant-specific T cells in these countries will be expected to confirm the findings in the present study. Here, we suggested a novel mechanism underlying the effects of multiple HLA alleles on disease progression. These data will contribute to our understanding of HIV-1 disease.

## MATERIALS AND METHODS

### Subjects.

We recruited 43 HLA-B*35:01^+^ Japanese individuals who were infected chronically with HIV-1 clade B and were antiretroviral therapy naive from the AIDS Clinical Center, National Center for Global Health and Medicine during January 2008 and January 2013. This study was approved by the ethics committee of the National Center for Global Health and Medicine and Kumamoto University. Informed consent was obtained from all individuals according to the Declaration of Helsinki. Peripheral blood mononuclear cells (PBMCs) were separated from whole blood. HLA types of HIV-infected individuals were determined by standard sequence-based genotyping.

### Cells.

721.221 cells expressing HLA-B*35:01 were previously generated by transfecting 721.221 cells with the HLA-B*35:01 gene (48), and the cells were maintained in RPMI medium containing 10% fetal calf serum (FCS; R10) and 0.15-mg/ml hygromycin.

### HIV-1 mutant clones.

HIV-1 strain NL4-3 carrying the SF2 strain-derived Nef135-143 epitope sequence (NL-4-3-Nef135Y) and its mutant virus carrying Nef135F (NL4-3-Nef135F) were generated previously (21).

### Bulk DNA sequencing and RNA deep sequencing of autologous virus.

Bulk sequencing of autologous virus was performed as described previously (15). To determine the frequencies of minor variants, we performed RNA sequencing using a MiSeq instrument (Illumina, USA) as described previously (49). Briefly, HIV-1 viral RNA was extracted from 1 ml of plasma samples from patient KI-705 (August 2009) by using a QIAamp UltraSens virus kit (Qiagen, UK) and linear acrylamide (Ambion, USA) instead of carrier RNA. DNase treatment was conducted with a Turbo DNA-free kit (Ambion). DNase-treated viral RNA samples were prepared for sequencing by using a NEBNext Ultra RNA library prep kit for Illumina (New England BioLabs, UK). Library preparation was performed with 5 μl of RNA without mRNA isolation. Double-stranded cDNA was purified by using AMPure XP beads (Beckman Coulter, USA). Library quality was assessed with an Agilent DNA high-sensitivity chip read on a bioanalyzer, Agilent 2100 (Agilent Genomics, UK). The prepared libraries were pooled for subsequent sequencing on an Illumina MiSeq platform using a 2 × 300-bp v3 kit (Illumina). MiSeq sequencing data were processed by an in-house pipeline using Sickle, Bowtie2, and SAMtools. Finally, variant calling was performed by using Varscan (http://varscan.sourceforge.net/).

### Establishment of epitope-specific T-cell clones or lines.

PBMCs from HLA-B*35:01^+^ individuals infected chronically with HIV-1 were stimulated for 14 days with an epitope peptide (100 nM) to induce peptide-specific bulk T cells. Epitope-specific T-cell clones were generated from bulk T cells using the limiting dilution method in 96-U plates, together with 200 μl of cloning mixture (5 × 10^5^ irradiated PBMCs from healthy donors, 1 × 10^5^ irradiated stimulator cells, and epitope peptides at a concentration of 100 nM in RPMI medium containing FCS, 200 U/ml of recombinant interleukin 2 [rIL-2], and 2.5% phytohemagglutinin [PHA]). In order to establish epitope-specific T-cell lines, PBMCs were stained with both phycoerythrin (PE)-conjugated YF9-tetramer and allophycocyanin (APC)-conjugated FF9-tetramer, followed by staining with fluorescein isothiocyanate (FITC)-anti-CD3 (Dako), Pacific Blue-anti-CD8 (BD Pharmingen, USA), and 7-aminoactinomycin D (7-AAD). Among CD3^+^CD8^+^7-AAD^−^ cells, YF9-specific, cross-reactive, or FF9-specific cells were sorted into a 96-well plate using a fluorescence-activated cell sorter (FACS) Aria I instrument. The sorted cells were stimulated with the corresponding epitope peptide and cultured for 2 to 3 weeks.

### Intracellular cytokine staining (ICS) assay.

721.221-B*35:01 pulsed with epitope peptides (0.1 to 10 nM) or 721.221-B*35:01 cells infected with HIV-1 virus were cocultured with the CTL clones or T-cell lines for 2 h at 37°C. Brefeldin A (10 μg/ml) was then added, and the cells were incubated for an additional 4 h. Cells were stained with allophycocyanin (APC)-labeled anti-CD8 MAb (Dako, Glostrup, Denmark) and fixed subsequently (4% paraformaldehyde), rendered permeable (0.1% saponin and 5% FCS), and stained intracellularly with fluorescein isothiocyanate (FITC)-labeled anti-interferon gamma (IFN-γ) MAb (BD Bioscience, USA). Data were analyzed with a FACS Canto II instrument.

### IFN-γ ELISPOT assays.

IFN-γ ELISPOT assays were performed as described previously (34). Briefly, PBMCs from HLA-B*35:01^+^ HIV-1-infected individuals were plated in the presence of 100 nM YF9 or RF10 epitope peptide or their variant FF9 or RF10-2F. The number of spots for each peptide-specific T-cell response was calculated by subtracting the number of spots in wells without peptides and standardizing to SFU/10^6^ CD8^+^ T cells. The mean + 4 SDs of the spots of samples from 9 HIV-1-naive individuals for the epitope peptides was 170 spots/10^6^ CD8^+^ T cells (34). Therefore, we defined a positive ELISPOT response as more than 200 spots/10^6^ CD8^+^ T cells.

### Tetramer binding assay.

HLA-class I peptide tetrameric complexes (tetramers) were synthesized as described previously (50). NefYF9 or NefFF9 peptides were added to the refolding solution containing the biotinylation sequence-tagged extracellular domain of the HLA-B*35:01 molecule and β_2_-microglobulin. The purified monomer complexes were mixed with PE- or APC-labeled streptavidin (Molecular Probes) at a molar ratio of 4:1. PBMCs or epitope-specific T-cell clones/lines were stained with YF9- and FF9-HLA-B*35:01 tetramers at 37°C for 30 min. The cells were then washed twice with R10 and then stained with FITC-anti-CD3 MAb (Dako), Pacific Blue-anti-CD8 MAb (BD Pharmingen), and 7-AAD (BD Pharmingen) at 4°C for 30 min. The cells were washed twice with R10. Data were analyzed with a FACS Canto II instrument. PBMCs unstained with tetramer were used as a negative control to determine the gating of the tetramer^+^ population. Background levels of the tetramer^+^ cells were calculated from the frequencies of tetramer^+^ cells of negative controls (mean + 2 SD < 0.01%). More than 0.01% of tetramer^+^ CD8^+^ cells were evaluated as positive values for tetramer staining. Frequencies of tetramer^+^ CD8^+^ T cells among PBMCs from HIV-1-uninfected individuals were similar to that among tetramer-unstained PBMCs from HIV-1-infected individuals as described previously (25).

### Flow cytometry analysis of perforin, granzyme B, T-bet, or inhibitory molecule expression in CD8^+^ T cells specific for Nef YF9/FF9 epitopes.

PBMCs from HLA-B*35:01^+^ individuals infected chronically with HIV-1 were stained with both PE-conjugated YF9-tetramer and APC-conjugated FF9-tetramer at 37°C for 30 min and then stained with APC/Fire750-conjugated anti-CD8 MAb (BioLegend), and 7-AAD (BD Pharmingen) at 4°C for 30 min. For an analysis of PD-1 and T-bet expression, the cells were stained with PE-Cy7-conjugated anti-PD-1 MAb (BioLegend) or the isotype control antibody at room temperature for 30 min and fixed subsequently (4% paraformaldehyde), made permeable (0.1% saponin and 5% FCS), and stained intracellularly with Alexa Fluor488-conjugated anti-T-bet MAb (BioLegend) or the isotype control antibody at room temperature for 30 min. For an analysis of perforin and granzyme B expression, the cells stained with tetramers, anti-CD8 MAb, and 7-AAD were fixed, rendered permeable, and stained intracellularly with PE-Cy7-conjugated anti-perforin MAb (BioLegend) and FITC-conjugated anti-granzyme B (BioLegend) or the corresponding IgG isotypes. For an analysis of 2B4, LAG-3, and CD160 expression, the cells were stained with tetramers and then stained with anti-CD8 MAb, 7-AAD, Brilliant Violet 421-conjugated anti-2B4 MAb (BioLegend), FITC-conjugated anti-LAG-3 MAb (BioLegend), and PE-Cy7-conjugated anti-CD160 MAb (BioLegend) or the corresponding IgG isotypes. Data were analyzed with a FACS Canto II instrument. The expression level of perforin, granzyme B, T-bet, or inhibitory molecules in tetramer^+^ CD8^+^ T cells from each individual was compared in terms of the ratio of mean fluorescence intensity (MFI). The ratio of MFI was calculated as MFI of molecule/that of isotype control.

### *Ex vivo* single-cell TCR repertoire analysis.

PBMCs from patient KI-705 were stained with both PE-conjugated YF9-tetramer and APC-conjugated FF9-tetramer and then stained with FITC-anti-CD3 (Dako), Pacific Blue-anti-CD8 (BD Pharmingen, USA), and 7-AAD. Among CD3^+^CD8^+^7-AAD^−^ cells, WT-specific, cross-reactive, or MT-specific cells were sorted into a 96-well plate by using a FACS Aria I instrument. Unbiased identification of TCR gene usage was assessed as described previously (51).

### Statistical analysis.

For the comparison of 2 groups in this study, a two-tailed Mann-Whitney test, the unpaired *t* test, or Wilcoxon signed-rank test was performed. The correlation between the frequencies of epitope-specific T cells and pVL was analyzed statistically by using the Spearman rank test. *P* values of <0.05 were considered to be statistically significant.
